# A sudden increase in heart rate during ablation of the right superior pulmonary venous vestibule is correlated with pain-relief in patients undergoing atrial fibrillation ablation

**DOI:** 10.1186/s12872-023-03121-1

**Published:** 2023-02-17

**Authors:** Ping Fang, Xianghai Wang, Meijun Zhang, Jichun Liu, Youquan Wei, Jinfeng Wang, Hao Yang, Xiangrong Xie, ShengXing Tang

**Affiliations:** 1 Anhui Medical University, Hefei, 230000 Anhui China; 2 Department of Cardiology, The First Affiliated Hospital (Yijishan Hospital) of Wannan Medical College, Wuhu, 230000 Anhui China; 3 Department of Intensive Care Medicine, The First Affiliated Hospital (Yijishan Hospital) of Wannan Medical College, Wuhu, 241001 Anhui China

**Keywords:** Atrial fibrillation, Circumferential pulmonary vein isolation, Pain, Ganglionated plexus, Conscious sedation

## Abstract

**Background:**

A sudden increase in heart rate (HR) during ablation of the right superior pulmonary venous vestibule (RSPVV) is often detected in patients undergoing circumferential pulmonary vein isolation (CPVI). In our clinical practices, we observed that some patients had few complaints of pain during the procedures under conscious sedation.

**Aim:**

We aimed to investigate whether there is a correlation between a sudden increase in HR during AF ablation of the RSPVV and pain relief under conscious sedation.

**Methods:**

We prospectively enrolled 161 consecutive paroxysmal AF patients who underwent the first ablation from July 1, 2018, to November 30, 2021. Patients were assigned to the R group when they had a sudden increase in HR during the ablation of the RSPVV, and the others were assigned to the NR group. Atrial effective refractory period and HR were measured before and after the procedure. Visual Analogue Scale (VAS) scores, vagal response (VR) during ablation, and the amount of fentanyl used were also documented.

**Results:**

Eighty-one patients were assigned to the R group, and the remaining 80 were assigned to the NR group. The post-ablation HR (86.3 ± 8.8 *vs.* 70.0 ± 9.4 b/min; *p* ≤ 0.001) was higher in the R group than in pre-ablation. Ten patients in the R group had VRs during CPVI, as well as 52 patients in the NR group. The VAS score [2.3 (1.3–3.4) *vs*. 6.0 (4.4–6.9); *p* ≤ 0.001)] and the amount of fentanyl used (107 ± 12 *vs.* 172 ± 26 ug; *p* ≤ 0.001) were significantly lower in the R group.

**Conclusion:**

A sudden increase in HR during the ablation of the RSPVV was correlated with pain relief in patients undergoing AF ablation under conscious sedation.

## Introduction

The radiofrequency catheter has been widely used for CPVI to treat AF worldwide.

A sudden increase in heart rate (HR) during ablation of the right superior pulmonary venous vestibule (RSPVV) is often detected in patients undergoing circumferential pulmonary vein isolation (CPVI) [1]. Radiofrequency catheter ablation of AF is a delicate procedure that requires patients to remain motionless for several hours. At the same time, AF ablation needs to burn the myocardium, and patients might suffer excruciating pain and experience anxiety. Therefore, anesthesia is essential for radiofrequency catheter AF ablation. The AF ablation is usually performed under general anesthesia, profound sedation, or conscious sedation, depending on the choice of the electrophysiological physician and the patient's general condition [2, 3]. Some clinical studies [4, 5] have shown that general anesthesia might improve the success rate of a single procedure and reduce the incidence of pulmonary venous conduction recovery. However, other studies have reached the opposite conclusions [6, 7]. The evident advantage of general anesthesia is that it can keep the patient still, improving catheter stability; the disadvantages are the increased risk associated with general anesthesia, increased medical costs, and the need for the involvement of anesthesiologists throughout the procedure. The most prominent advantage of general anesthesia is that patients do not feel pain during the procedure. For AF ablation, the advantages of conscious sedation are no need for an anesthesiologist, reduced anesthesia risk and economic costs, and shortened procedural time. The main disadvantage of conscious sedation is that some patients might experience severe pain during procedures. In our electrophysiological catheter laboratory, most patients undergoing AF ablation are under conscious sedation, and only a few patients unable to cooperate are given general anesthesia. In our clinical practice, we found that some patients experience no obvious pain during the procedures, while others experience excruciating pain. Nevertheless, few studies have explored whether the suddenly accelerated HR caused by the ablation of the RSPVV is correlated with diminished pain in patients during CPVI. Therefore, in this study, we investigated whether a sudden increase in HR caused by AF ablation of the RSPVV is correlated with pain relief in patients under conscious sedation.

## Methods

### Study population

This is a prospective observational study, and was approved (No.LS201812) by the Institutional Review Board (IRB) of the First Affiliated Hospital (Yijishan Hospital) of Wannan Medical College. In total, 161 consecutive paroxysmal AF patients undergoing the first ablation at the Yijishan Hospital were enrolled from July 1, 2018 to November 30, 2021. Patients with previous ablation history, an anterior–posterior diameter of the left atrium significantly enlarged by more than 60 mm, with left atrial thrombus, or a history of previous cardiac surgery were excluded. Enrolled patients were divided into R and NR groups based on whether they experienced (R) or not (NR) a sudden increased HR during the ablation of the RSPVV. If patients were taking some anti-arrhythmic medications, the procedures should be performed after 5 half-lives of these drugs had been discontinued. The post-procedural decision to resume the anti-arrhythmic depended on the patient's condition.

### Definition of sudden increased HR

The definition of sudden increased HR means that: patient experiences a sudden increase in HR of more than 10 beats/min within 10 s when ablating the RSPVV.

### Anesthesia and ablation protocols

**Fig. 1:**
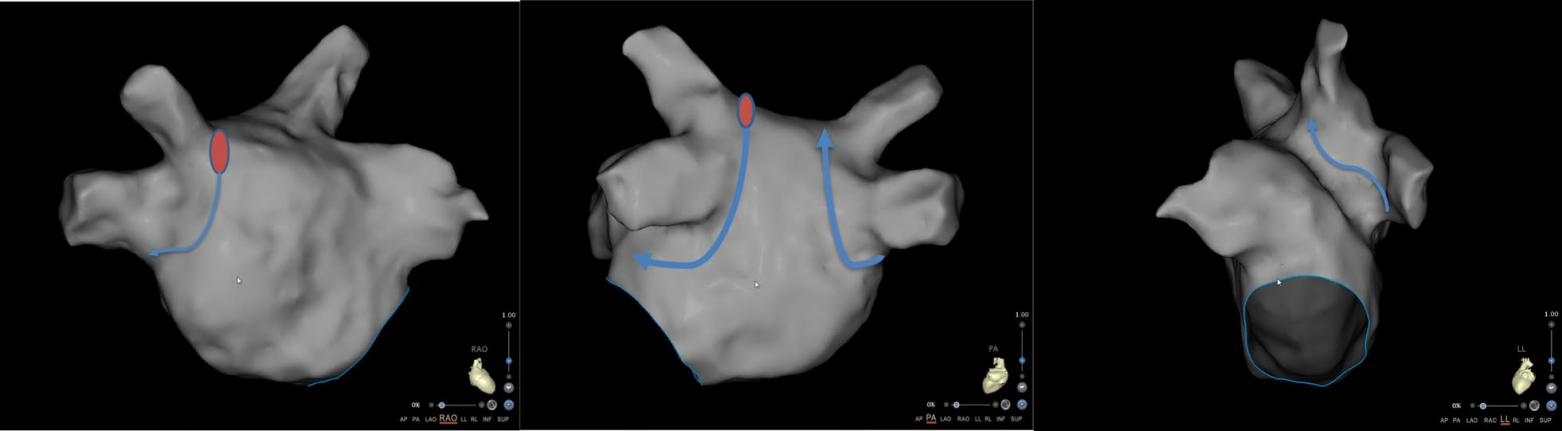
Ablation initiation region and sequence. The red areas indicate the initial site of ablation; the blue arrows indicate the ablation sequence

**Fig. 2:**
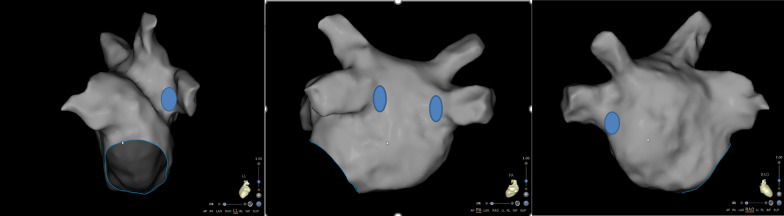
Four sites needed VSA scoring. The blue areas indicate the sites needed VSA scoring

All patients underwent CPVI under conscious sedation through intravenous administration of fentanyl and midazolam. At the beginning of the procedure, before peripheral vascular puncture, we started a continuous infusion of midazolam (0.03–0.2 mg/kg/h) to achieve a Richmond Agitation-Sedation Scale (RASS) [8] between − 1 and 1. Femoral or left subclavian veins were cannulated with local anesthesia using lidocaine (2%). A quadripolar electrode catheter (Biosense Webster, Inc, Irvine, CA) was introduced into the right ventricle through the left femoral vein, and a decapolar electrode catheter (Biosense Webster, Inc, Irvine, CA) was positioned in the coronary sinus through the left femoral or left subclavian vein. After the transseptal puncture, we started pumping fentanyl intravenously (1 ug/kg/h). When patients felt severepain (VAS score ≥ 6) during the ablation, the dosage of fentanyl was increased to 2 ug/kg/h. If the patients had only mild pain (VAS score ≤ 3) during subsequent ablation, the dose of fentanyl was reduced to 1 ug/kg/h. Fentanyl infusion was suspended if patients experienced intolerable nausea, vomiting, hypotension, or respiratory depression during fentanyl infusion. If the patient experiences excruciating pain (VAS score ≥ 8) after suspension of fentanyl pumping, 5 mg morphine injection may be given subcutaneously. In all patients, fentanyl pumping was stopped immediately after CPVI was completed. Ablation (Navistar Thermocool Smart Touch, Biosense Webster, Inc, Irvine, CA) and mapping catheters (PantaRay, Biosense Webster, Inc, Irvine, CA) were introduced into the left atrium via the right femoral vein. After the atrial septal puncture, heparin was administered intravenously at 100 IU/kg. Patient’s activated clotting time (ACT) was measured every 30 min, and extra boluses were given to maintain the ACT between 330 and 350 s. The left atrium was mapped using the PantaRay catheter under the guidance of the CARTO-3 System (Biosense Webster, Inc, Irvine, CA). The ablation started from the right superior pulmonary venous vestibule. The ablation of the left pulmonary vein initiated from the roof of the left atrial posterior wall. The exact sites are shown in Fig. 1. All procedures were performed under the ablation index (AI) guidance. The AI was set at 380–400 for the left atrium posterior wall and 450–480 for the left atrium anterior wall. The specific sequence of ablation is also shown in Fig. 1. The Smart Touch (ST) ablation catheter was used in the power-controlled mode. The power limit of the left atrium posterior wall was 30 W and the anterior wall 35 W with a temperature limit of 43 °C. The ST catheter was continuously irrigated with saline at 17 mL/min, and its contact force was maintained within 5–20 g during the CPVI. Atrial effective refractory period (AERP) and HR were recorded before and post-ablation using the multichannel recorder (Bard Electrophysiology, Lowell, MA). The AERP was measured using the decapolar electrode catheter electrodes 1–2 at distal coronary sinus with S1S2 stimulus at twice the diastolic threshold by a decrement of 5 ms. The S1S1 cycle length started from 550 ms, and the coupling S2 interval started from 500 ms. The pain was evaluated using the Visual Analogue Scale (VAS) [9] during CPVI procedures. The VAS score was evaluated when ablating the right inferior pulmonary vein posterior and anterior walls, left inferior pulmonary vein posterior and anterior walls in our clinical observations, these four sites (pain-prone areas) are common sites where patients feel serious pain during CPVI. The mean VAS (VASm) score represented the average score of these four sites. The specific sites requiring VAS scoring are shown in Fig. 2. Vagal responses (VRs) were recorded and defined as an R-R interval extended by more than 50%, atrioventricular block, and transient ventricular asystole. When patient^`^s HR fell below 40 beats per minute, temporary ventricular pacing would be given. The amount of fentanyl used during the procedure was calculated, and fentanyl-related side effects were documented. Procedure duration, complications, ablation time, and intraprocedural X-ray exposure time were documented.

### Statistical analysis

The sample size estimated for this study was based on our previous clinical observation. About 50 percent of patients experienced a sudden increase in heart rate during ablation of the right pulmonary vein. Patients who did not experience a sudden increase in heart rate had an average VAS score of 5.9 and those who experienced a sudden increase in heart rate had an average VAS score of 2.7. A study with 146 patients would have 90 percent power to detect a 3.2 decreased in VAS score in the R group compared with the NR group, on the basis of an α level for each comparison of 0.05. We increased the sample size by 10 percent, so we enrolled 161 patient in this study.All statistical analyses were performed using SPSS version 25.0 (Inc. Chicago, IL). Continuous variables with normal distribution are presented as means ± standard deviation (SD) and were compared using the unpaired two-sample or paired Student's t-test. Non-normally distributed continuous variables are presented as medians (25% quartile, 75% quartile) and were compared using the Wilcoxon rank-sum test. Categorical variables are expressed as counts or percentages, and the χ^2^ test or Fisher’s exact test was used to compare groups. A *p* < 0.05 was considered statistically significant.

## Results

### Patients^,^ characteristics

In total, 161 patients undergoing the first AF ablation were enrolled, in whom 81experienced sudden increase in HR during the ablation of the RSPVV during CPVI (R group), and the event was free in another 80 patients (NR group). The baseline characteristics were not different in these patients (*p* > 0.05), and are shown in Table 1.

**Table 1:** Baseline characteristics of patients

	R group	NR group	*p* value
Age, y	63.0 ± 9.0	61.7 ± 10.0	0.379
CHA2DS2-VASc score	2 (1–3)	2 (1–3)	0.875
Sex, male, n (%)	39 (48.0)	36 (45)	0.689
BMI, kg/m^2^	22.5 ± 2.3	22.8 ± 2.4	0.529
Smoking, n (%)	10 (12.3)	11 (13.8)	0.791
Comorbidities			
Hypertension, n (%)	43 (53.1)	46 (57.5)	0.573
Diabetes mellitus, n (%)	7 (8.6)	11 (13.8)	0.304
Echocardiography measures			
LVEF, n (%)	63.4 ± 4.1	62.1 ± 4.5	0.052
LA diameter, mm	39.2 ± 5.1	38.8 ± 5.1	0.620
Medications before catheter ablation			
β-Blocker, n (%)	15 (18.5)	11 (13.8)	0.411
ACEI or ARB, n (%)	14 (17.3)	14 (17.5)	0.971
Amiodarone, n (%)	6 (7.4)	9 (11.3)	0.402
Propafenone, n (%)	8 (9.1)	6 (5.6)	0.593

*BMI* Body mass index; *LVEF* Left ventricular ejection fraction; *LA* Left atrium; *ACEI* Angiotensin converting enzyme inhibitor; *ARB* Angiotensin receptor blocker

### Procedural parameters

There were no significant differences between the two groups regarding the ablation time of anterior wall [(16.5 ± 2.7) min *vs*. (17.0 ± 2.7) min; *p* = 0.228)], ablation time of posterior wall [(10.8 ± 2.2)min *vs*. (11.3 ± 1.8) min; *p* = 0.102)], ablation time of pain-prone areas [(3.2 ± 0.4) min *vs*. (3.1 ± 0.4) min; *p* = 0.053)], X-ray exposure time [(7.4 ± 1.0) min *vs*. (7.4 ± 1.2) min; *p* = 0.776)], and procedure time [(148 ± 31) min *vs.* (151 ± 27) min; *p* = 0.585)]. The first-pass isolation rate for right pulmonary vein (RPV) was 85% (137/161), and for left pulmonary vein (LPV) was 80% (129/161) in the two groups. The most acute reconnection sites were posterior wall in RPV and LPV. The patients complained of the same level of pain as the initial ablation at the second round of ablation for the acute reconnection sites. Ten patients in the R group experienced VR during CPVI compared to 52 patients in the NR group. Furthermore, only one patient in the R group required temporary ventricular pacing support when they developed VR during CPVI, compared to 22 patients in the NR group. Only one patient in each group had peripheral vascular complications. Further details are presented in Table 2.

**Table 2:** Procedural parameters of the two groups

	R group	NR group	*p* value
Ablation time of different areas of left atrium, min			
Posterior wall	10.8 ± 2.2	11.3 ± 1.8	0.102
Anterior wall	16.5 ± 2.7	17.0 ± 2.7	0.228
Pain-prone areas	3.2 ± 0.4	3.1 ± 0.4	0.053
First-pass isolation rate of RPV, n (%)	70 (86)	67 (84)	0.634
First-pass isolation rate of LPV, n (%)	66 (81)	63 (79)	0.664
Patients had VR, n (%)	10 (12.3)	52 (65.0)	0.000
Temporary pacing, n (%)	1 (1.2)	22 (27.5)	0.000
The mean X-ray exposure time, min	7.4 ± 1.0	7.4 ± 1.2	0.776
The mean procedure time, min	148 ± 31	150 ± 27	0.585
Complications, n			
Tamponade	0	0	–
Peripheral vascular complications	1	1	1.000
Pneumothorax	0	0	–

n stands for the number; min indicates minute

### VASm score, fentanyl usage, and fentanyl-related side effects

There were two patients in the R group and six patients in the NR group who stopped pumping fentanyl during the procedures due to hypotension and respiratory depression. These patients did not experience unbearable pain during subsequent ablation and were not given morphine for analgesia. During the CPVI procedure, the subjective pain sensation of patients in the R group was significantly lower than that in the NR group. The VASm score [2.3 (1.3–3.4) *vs.* 6.0 (4.4–6.9); *p* = 0.000] was lower than that in the NR group. Moreover, the intraprocedural dosage of fentanyl (107 ± 12 *vs.* 172 ± 26 µg; *p* = 0.000) in the R group was significantly reduced compared to the NR group. Common side effects of fentanyl (nausea and vomiting) (4 *vs.* 28; *p* = 0.000) were significantly different between the R and NR groups. However, there was no significant difference in hypotension (1 *vs.* 6; *p* = 0.064) and respiratory depression (1 *vs.* 2; *p* = 0.620) caused by fentanyl between the two groups. The details are shown in Table 3.

**Table 3:** Procedural VASm score, the amount of fentanyl used, and fentanyl-related side effects

	R group	NR group	*p* value
VASm score, n	2.3 (1.3–3.4)	6.0 (4.4–6.9)	0.000
Amount of fentanyl used, ug	107 ± 12	172 ± 26	0.000
Fentanyl-related side effects			
Nausea and vomiting, n (%)	4 (4.9)	28 (35.0)	0.000
Hypotension, n (%)	1 (1.2)	6 (7.5)	0.064
Respiratory depression, n (%)	1 (1.2)	2 (2.5)	0.620

*n* Number

### Electrophysiological parameters

The HR in the R group was significantly increased after ablation (post-ablation) compared with that before ablation (pre-ablation) (70.0 ± 9.4 *vs.* 86.3 ± 9.8 b/min; *p* = 0.000), while the HR in the NR group was not significantly changed during the procedures (72.4 ± 9.9 *vs.* 72.4 ± 8.7 b/min; *p* = 0.979). The pre-ablation atrial effective refractory period (AERP) (219 ± 26 *vs.* 225 ± 25 ms; *p* = 0.307) did not differ between the two groups. The patients in the R group showed prolonged AERP (242 ± 25 *vs.* 219 ± 26 ms; *p* ≤ 0.001) after CVPI procedures compared to pre-ablation. However, pre-ablation and post-ablation AERPs (223 ± 24 *vs.* 223 ± 23 ms; *p* = 0.907) in the NR group did not significantly change (Table 4). In addition, in the routine electrophysiological study after the completion of CPVI, atrioventricular nodal reentrant tachycardia (AVNRT) was induced in one patient in the R group, and atrioventricular reentrant tachycardia (AVRT) was induced in one patient in the HR group. Both patients were given successfully radiofrequency ablation.

**Table 4:** Electrophysiological parameters of the two groups

	R group	NR group	*p* value
Pre-ablation			
HR, b/min	70.0 ± 9.4	72.4 ± 9.9	0.124
AERP, ms	219 ± 26	223 ± 24	0.307
Post-ablation			
HR, b/min	86.3 ± 9.8	72.4 ± 8.7	≤ 0.001
AERP, ms	242 ± 25	223 ± 23	≤ 0.001
P_H_ value	≤ 0.001	0.979	
P_A_ value	≤ 0.001	0.907	

*b/min* Beat/minute; P_H_ and P_A_ values show the significances of the HR and the AERP between pre- and post-ablation in the same group, respectively

## Discussion

Radiofrequency ablation has been widely used to treat AF. Ablation of AF needs to burn the atrial muscles, and patients might suffer excruciating pain. Therefore, anesthesia is essential for radiofrequency catheter AF ablation. In patients undergoing catheter ablation of AF under conscious sedation, some still experience unbearable pain during the procedure, while others have no evident pain sensation. To the best of our knowledge, there are few studies to investigate the influencing factors of pain during radiofrequency catheter ablation in patients with AF under conscious sedation. In the present study, we found that the patients who experienced sudden HR acceleration during the ablation of the RSPVV had significantly lower VAS scores than those who did not. We also observed that patients with a sudden increase in HR during the ablation of the RSPVV had fewer VRs, significantly increased mea post-procedure HR, significantly prolonged AERP, reduced amounts of analgesic drugs used during procedures, and reduced side effects associated with analgesics.

Sudden HR increase during ablation of the RSPVV is common during the CPVI in our clinical practice. Thus, we hypothesized that this phenomenon occurs because the ablation creates a coincidental modulation of the cardiac ganglionated plexus (GP). Anatomical studies [10–12] have shown that most heart's GPs are located at the junctions between the atrium and pulmonary veins. There are four major GPs in and near the left atrium. The right anterior GP (RAGP) is located between the right superior pulmonary vein and right atrium, the right inferior GP (RIGP) is located between the right inferior pulmonary vein and the right atrium, the left superior GP (LSGP) is located between the left superior pulmonary vein and the left atrium, and the left inferior GP (LIGP) is located between the left inferior pulmonary vein and the left atrium. Hu et al. [1] found that patients often experience a sudden increase in heart rate during the ablation of the RAGP from the endocardial of the left atrium but not with ablation of the other ganglion plexuses (GPs) elsewhere in the left atrium.

Some studies [13–15] have shown that some nerve fibers of cardiac GPs are distributed to the sinoatrial node and the atrioventricular node region. Thus, cardiac GPs can regulate atrioventricular node and sinoatrial function via these nerve fibers. Chiou et al. [16] demonstrated that most vagal fibers to the atrium, sinus and atrioventricular node travel through the RAGP area. Hu et al. [17] also found that ablating the RAGP first can significantly reduce the VRs during subsequent ablation. The RAGP is considered the “integration center” between extrinsic and intrinsic cardiac autonomic nervous systems. In the present study, based on cardiac anatomy, we speculated that the sudden HR acceleration in R group during ablation of the RSPVV was due to RAGP modification by radiofrequency ablation and the RAGP did not receive modification or the modification was insufficient during CPVI in non-R group. Patients in R group experienced less pain during CPVI than those without a sudden heart rate increase. In the present study, we first ablated the right pulmonary vein, yet 39% (62/161) of the patients experienced VRs during the procedures, which was relatively higher than the incidence described in previous report [17]. We speculated that higher proportion of VR in our study may be associated with two factors. First, the AI value in the right pulmonary vein vestibular was lower (440–480) in our study; Next, we did not perform wide area circumferential ablation circle for the patients included in current study. The factors aforementioned may lead to insufficient modification of the RAGP during CPVI.

AF ablation can be painful if patients are under conscious sedation, and sometimes it is difficult to relieve the pain with analgesic drugs. Aryana et al. [18] found that the main reason for pain during CPVI is that the ablation sites are close to the esophagus, and the pain sites during ablation are mostly in the posterior left atrial wall, while the anterior left atrial wall is absent. However, Attanasio et al. demonstrated [19] that there is no correlation between the pain sensation area and the esophagus location. They found that pain reactions often occur in the area of the left superior pulmonary vein (LSPV) ostium because more afferent nociceptive pain fibers are located there. Cardiac GPs and their afferent nociceptive pain fibers have an important role in pain perception within the heart and are widely distributed throughout the whole left atrium, especially in the pulmonary vein ostium [20]. We observed that during the CPVI procedure, the sites of severe pain in patients were consistent with the anatomical distribution of the cardiac GPs described above. Considering the integration center of the intrinsic cardiac GPs, RAGP has synaptic connections with other GPs in the heart and synaptic connections with the extrinsic cardiac autonomic nervous system [13, 17, 21]. Therefore, RAGP can integrate cardiac nociceptive nerve afferents signal from other GPs. We hypothesized that in patients with a sudden increase in HR during ablation of the RSPVV, intraprocedural pain-relief is due to modification or destruction of the RAGP.

The RAGP is generally located in the epicardial fat pad. The fat pad thickness is different in patients, and its location also has anatomical variations, and nearly half of the patients did not experience a sudden increase in HR during the ablation of the RSPVV. Here, patients with sudden rate acceleration also had a higher HR after the procedure, consistent with Hu`s et al. They found that the increased HR of these patients could be sustained for a long time.

The amounts of cholinergic fibers distributed in each cardiac GP differ, and the cholinergic fibers account for a relatively high proportion [22]. When cardiac GPs are stimulated, the parasympathetic nerve activity usually increases, and the patient develops VR. Herein, we found that the number of VRs during CPVI in the R group patients was significantly lower than in the NR group. When the function of the RAGP as an integration center is modified or destroyed, it will inevitably cause the abnormality of other cardiac GPs, consistent with the significantly reduced VRs in the R group during the CPVI procedure.

Previous studies [23, 24] have demonstrated that cardiac GP regulates the effective refractory period of cardiomyocytes by regulating the open state of potassium, sodium, and other ion channels via its abundant nerve fibers. Here, we found that the AERPs of patients in the R group was more prolonged than pre-ablation, while there was no significant change in patients in the NR group. This phenomenon might also be related to RAGP modification.

### Limitations

Our study also has limitations. First, this is a single center small sample clinical observational study, and multi-center and large sample cohorts are needed in the future. Second, the VSA score used in this study is a subjective pain assessment scale and might be biased from the real situation. Next, it is better to determine the location of RAGP via high frequency stimulation firstly, and perform the RAGP ablation accurately. Considering GP ablation is not a routine ablation strategy for AF currently and we didn't have a lot of experience with GP ablation, so we did not give high frequency stimulation to identify the RAGP. Finally, no follow-up had been conducted on recurrence rate of AF in the two groups.

## Conclusion

In summary, we showed that a sudden increase in HR during the ablation of the RSPVV is related to pain relief in patients undergoing CPVI under conscious sedation. We also observed that patients in the R group had reduced VRs and analgesic drug consumption during the procedures. The AERPs of the patients was prolonged, and the heart rate still accelerated in the R group compared to pre-ablation. We hypothesized that this phenomenon might be related to modifying the RAGP by radiofrequency catheter ablation. Therefore, to reduce the pain of AF patients during CPVI under conscious sedation, we might first consider ablating RAGP from the left atrial endocardium. Nevertheless, our current results still need to be confirmed by multi-center and large-sample clinical trials.

## Data Availability

The data involved in this study are available in an unidentified form from the corresponding author on reasonable request.
